# Dendritic cell activation enhances anti-PD-1 mediated immunotherapy against glioblastoma

**DOI:** 10.18632/oncotarget.25061

**Published:** 2018-04-17

**Authors:** Tomas Garzon-Muvdi, Debebe Theodros, Andrew S. Luksik, Russell Maxwell, Eileen Kim, Christopher M. Jackson, Zineb Belcaid, Sudipto Ganguly, Betty Tyler, Henry Brem, Drew M. Pardoll, Michael Lim

**Affiliations:** ^1^ Department of Neurosurgery, Johns Hopkins University School of Medicine, Baltimore, MD, USA; ^2^ Department of Cancer Immunology, Johns Hopkins University School of Medicine, Baltimore, MD, USA

**Keywords:** PD-1, immunotherapy, glioblastoma, dendritic cells, antigen presentation

## Abstract

**Introduction:**

The glioblastoma (GBM) immune microenvironment is highly suppressive as it targets and hinders multiple components of the immune system. Checkpoint blockade (CB) is being evaluated for GBM patients. However, biomarker analyses suggest that CB monotherapy may be effective only in a small fraction of GBM patients. We hypothesized that activation of antigen presentation would increase the therapeutic response to PD-1 blockade.

**Results:**

We show that activating DCs through TLR3 agonists enhances the anti-tumor immune response to CB and increases survival in GBM. Mice treated with TLR3 agonist poly(I:C) and anti-PD-1 demonstrated increased DC activation and increased T cell proliferation in tumor draining lymph nodes. We show that DCs are necessary for the improved anti-tumor immune response.

**Conclusions:**

This study suggests that augmenting antigen presentation is an effective multimodal immunotherapy strategy that intensifies anti-tumor responses in GBM. Specifically, these data represent an expanded role for TLR3 agonists as adjuvants to CB.

**Methods:**

Using a preclinical model of GBM, we tested the efficacy of combinatorial immunotherapy with anti-PD-1 and TLR3 agonist, poly(I:C). Characterization of the immune response in tumor infiltrating immune cells and in secondary lymphoid organs was performed. Additionally, dendritic cell (DC) depletion experiments were performed.

## INTRODUCTION

Glioblastoma (GBM) is the most common primary brain tumor in adults. Despite surgical resection, chemotherapy, and radiotherapy survival and prognosis are grim with median survival hovering at approximately 15 months with standard of care [1–4]. Immune checkpoint blockade (CB) has yielded durable responses in multiple tumor types and immune checkpoint blocking antibodies are being actively investigated in GBM. In particular, Programmed Cell Death 1 (PD-1) seems to play a critical role in preventing tumor rejection and PD-1 blocking antibodies have shown impressive activity in melanoma, NSCLC, and bladder cancer, among others.

Checkpoint molecules are high jacked by tumors to evade the immune surveillance. Antibodies targeting these molecules have recently been approved by the Food and Drug Administration (FDA) for the treatment of multiple cancers [5]. Anti-PD-1 therapy restores the effector function of exhausted T cells and promotes anti-tumor immune response [5–8]. PD-1 is expressed on T cells and its known ligands, PD-L1 and PD-L2, are expressed by tumor cells, antigen presenting cells, and tumor associated myeloid cells [7, 9, 10]. However, comprehensive biomarker analyses suggest that CB monotherapy may be effective only in a small subset of GBM patients [11].

The role of myeloid cells in the antitumor immune response in the context of CB is not as well-defined. Traditionally, myeloid cells such as dendritic cells process antigens and then present them in the draining lymph nodes. Antigen presentation is indispensable to generate T cell responses against cellular antigens, including neoantigens generated during the process of tumorigenesis [12]. In the central nervous system (CNS); however, the mechanisms of antigen presentation are a topic of ongoing investigation [13]. With an increasing focus on the relationship between biomarkers such as microsatellite instability, mutational burden, and responses to CB [14, 15], understanding the mechanisms of antigen presentation in the setting of immune CB will be critical to effective clinical translation. We assessed the role of myeloid cells in mediating CNS antitumor immunity using (polyriboinosinic-polyribocytidic acid) (Poly(I:C)) as a tool to promote maturation of dendritic cells (DCs) [16] in the setting of PD-1 blockade.

We hypothesized that dendritic cells were responsible for antigen presentation primarily in the deep draining cervical lymph nodes and that activating antigen presentation resulted in enhanced response to CB. Our results suggest that antigen presentation plays a crucial role in generating an antitumor immune response.

## RESULTS

### TLR3 agonist enhances activation of dendritic cells (DCs)

**Figure 1:**
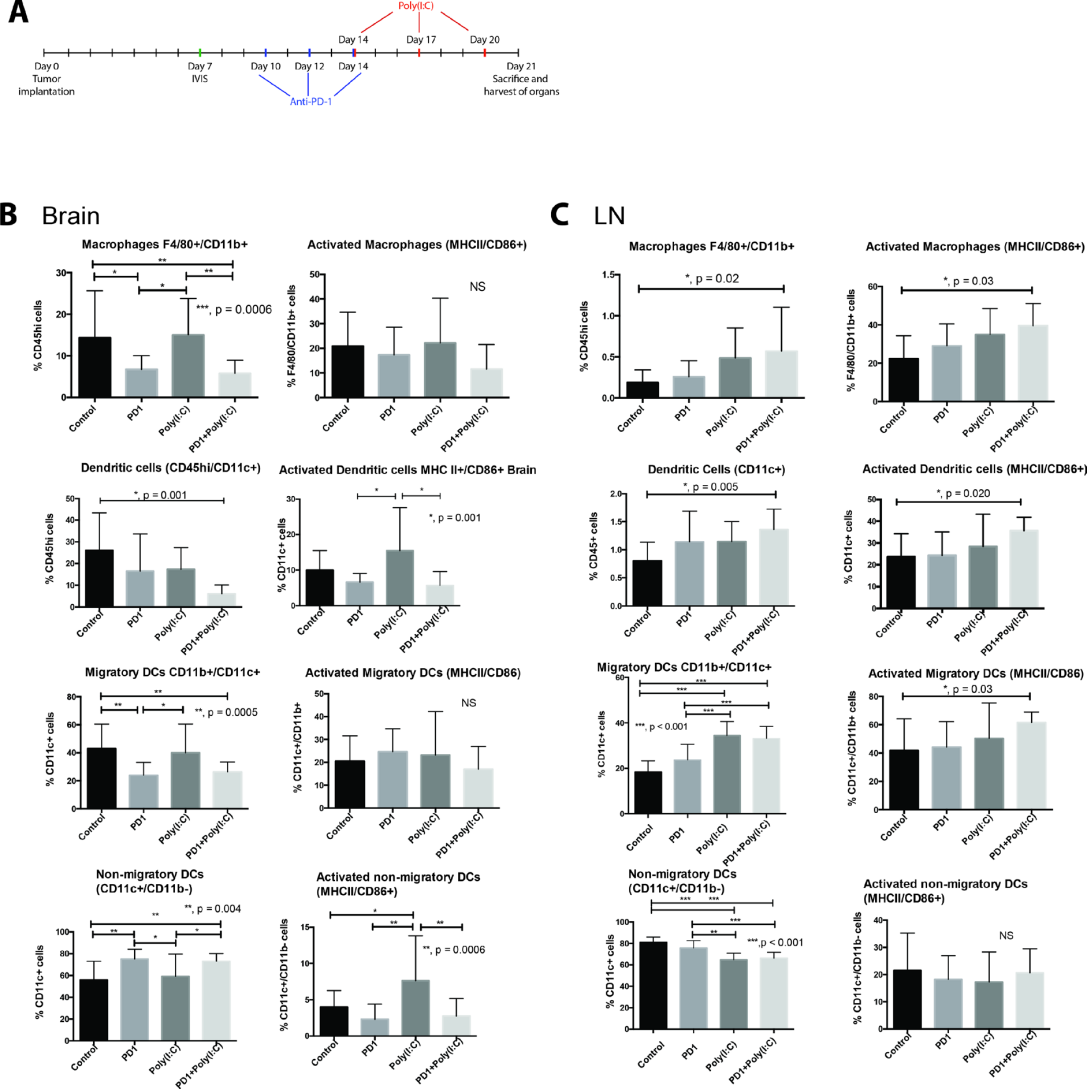
TLR3 agonist enhances activation of dendritic cells (DCs) (**A**) Treatment schedule. Mice that received an intracranial injection with GL261-luciferase cells were imaged with bioluminescence (IVIS) on day 7 to ensure similar tumor burden in all mice, after IVIS imaging, mice were randomized to each treatment group. Anti-PD-1 treatment was given on days 10, 12, and 14 via intraperitoneal (i.p.) injection. Poly(I:C) was given on days 14, 17, and 20 via i.p. injection. (**B**–**C**) Evaluation of macrophage (F4/80^+^/CD11b^+^), Dendritic cells (DC, CD11c^+^), migratory DCs (CD11c^+^/CD11b^+^), and non-migratory DCs (CD11c^+^/CD11b^−^) and their activation status in the brain (B) and draining lymph node (C). Bar charts show percentage of cells gating on CD45^+^ and gating on CD45^hi^ in the brain. We observed an increase in the percentage and activation of macrophages and DCs in deep cervical lymph nodes, particularly in the case of migratory DCs. Interestingly, the percentage of macrophages and DCs infiltrating the brain tumor decreased in the anti-PD-1+poly(I:C) group as compared to control mice. Data are represented as mean ± SEM. All experiments repeated in triplicate with ≥5 mice per arm. *P*-values were determined by ANOVA, and, ^*^denotes statistical significance (*p* < 0.05).

Using an orthotopic mouse glioma model, we investigated the immune response after treatment with TLR3 agonist Poly(I:C) alone and in combination with anti-PD-1. We studied the number and activation status of tumor infiltrating myeloid cells, in draining lymph nodes, and in spleen at the end of the treatment schedule (Figure 1A). In brain tumor infiltrating myeloid cells the percentage of CD45^+^ cells that were macrophages (F4/80^+^/CD11b^+^) was significantly lower in anti-PD-1 and anti-PD-1+Poly(I:C) treated mice as compared with control mice or Poly(I:C) alone treated mice (Figure 1B). There were no statistically significant differences in activation of macrophages (MHCII^+^/CD86^+^). The percentage of CD45^+^ cells that were dendritic cells (DCs) (CD45hi/CD11c^+^) was significantly lower in mice treated with anti-PD-1+Poly(I:C) as compared with control mice (*p =* 0.001) (Figure 1B). Treatment with Poly(I:C) resulted in a greater percentage of activated DCs (MHCII+/CD86+) compared to anti-PD1 and anti-PD1+poly(I:C) groups (*p =* 0.001). Further stratification of DC populations revealed notable differences. Treatment with either anti-PD1 or anti-PD1+Poly(I:C) revealed significantly fewer CD11c^+^/CD11b^+^ (migratory DCs) compared to control or poly(I:C) groups (*p =* 0.0005). Treatment with either anti-PD-1 alone or anti-PD-1+poly(I:C) resulted in an increase in CD11c^+^/CD11b^−^ (non-migratory DCs) compared to control or poly(I:C) treatment, suggesting this change was secondary to anti-PD-1 treatment (*P =* 0.004). Poly(I:C) treatment resulted in significantly higher activation of CD11c^+^/CD11b^−^ cells as compared to the all other experimental groups (*p =* 0.004). There were no significant changes in activation status of CD11c^+^/CD11b^+^ cells. These results indicate that poly(I:C) treatment is effective in enhancing resident DC activation in the brain tumor microenvironment.

Next we investigated the microglia compartment since previous reports show that TLR3 activation in cultured microglia induces their activation [17]. Interestingly, the percentage of activated microglia (CD45^lo/^MHCII^+^/CD86^+^) CD45lo cells was significantly lower in the anti-PD1+Poly(I:C) group as compared to those in the control group (data not shown *p =* 0.022). Previous reports suggest expression of CD11c on microglial cells identifies a unique population with antigen presenting capabilities [18]. There were no differences in the percentages of CD45^lo^/CD11c^+^ cells among the different treatment groups and treatment with anti-PD1+Poly(I:C) resulted in a lower percentage of activated CD45^lo^/CD11c^+^ cells compared to the control (*p =* 0.027). There were no differences in percentage of tumor infiltrating CD45^+^/CD11b^+^/Ly6g^+^ cells (data not shown).

We also investigated changes in myeloid cells in the deep cervical lymph nodes designated as tumor draining lymph nodes. Treatment with anti-PD1+poly(I:C) resulted in an increase in macrophages and the percentage of activated macrophages compared to control (Figure 1C, *p =* 0.02 and *p =* 0.03 respectively). Additionally, treatment with anti-PD1+Poly(I:C) resulted in an increase in DCs and activated DCs compared to control (Figure 1C, *p =* 0.005 and *p =* 0.020 respectively). The percentage of migratory DCs increased significantly in the Poly(I:C) and anti-PD1+Poly(I:C) as compared to control and anti-PD1 (Figure 1C, *p <* 0.001). Activation status revealed a statistically significant increase in the anti-PD1+poly(I:C) group only when compared to the control group (*p =* 0.03). There was a decrease in the percentage of non-migratory DCs in the poly(I:C) and anti-PD1+poly(I:C) groups as compared to the control and anti-PD1 groups (Figure 1C, *p <* 0.001). There were no differences in the activation status of non-migratory DCs in the lymph node. No significant changes were seen in the spleen. Thes data suggest that TLR3 activation leads to increased activation of migratory DCs, which may lead to increased tumor antigen presentation in the tumor draining lymph nodes.

Overall, these data show that TLR3 agonist poly(I:C) results in enhancement in numbers and activation of myeloid cells, specifically macrophages and migratory dendritic cells in in the tumor and tumor-draining lymph nodes suggesting that there is an enhanced potential for antigen presentation and formation of an effective immune response.

### TLR3 agonist decreases percentage of Tregs infiltrating brain tumors and promotes tumor infiltration with effector T cells

**Figure 2:**
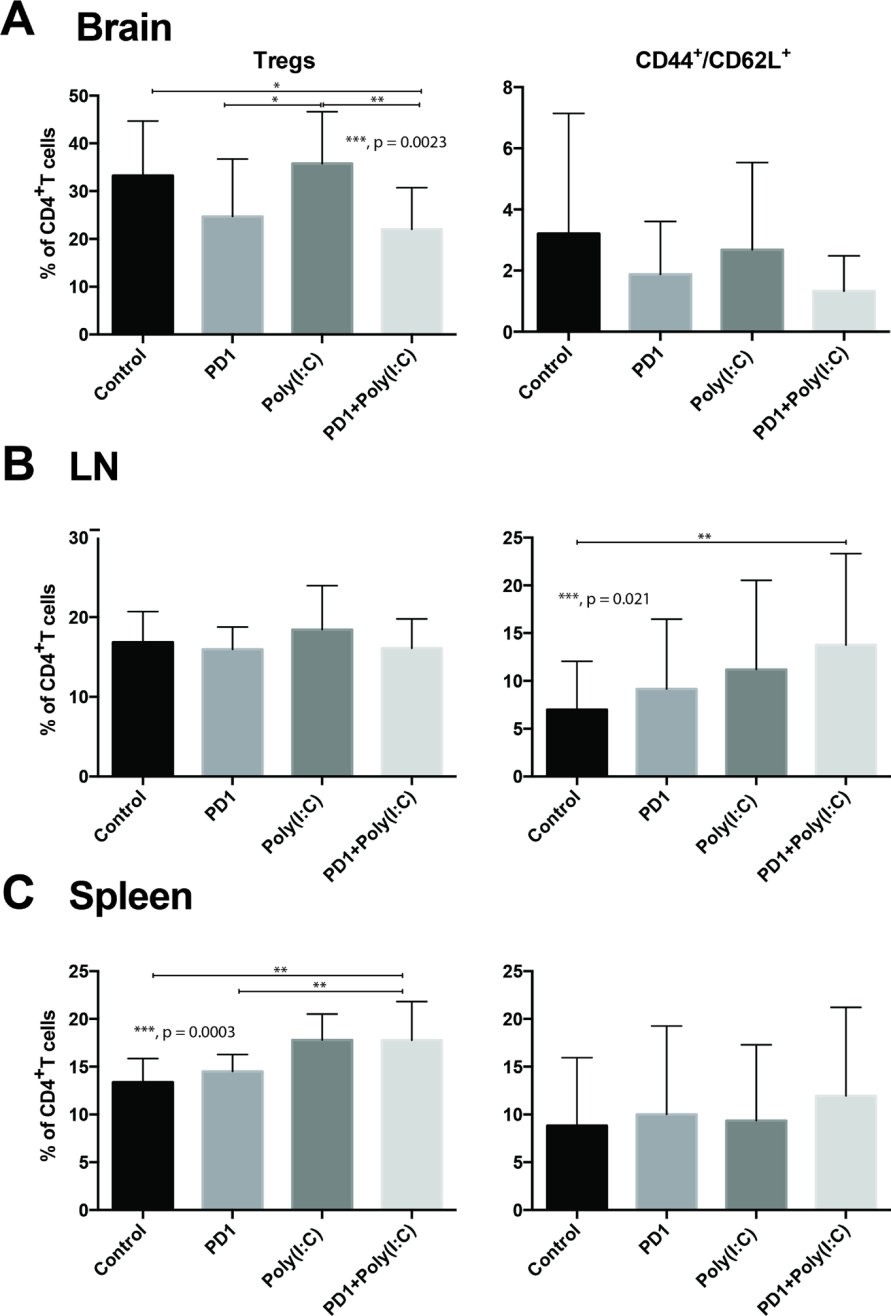
Effect of PD-1 blockade in addition to TLR3 agonist on T cells Bar charts demonstrating changes in T cell populations in different treatment groups. Anti-PD-1+poly(I:C) decreases percentage of CD4^+^FoxP3^+^ Tregs infiltrating the brain tumor and slightly increases infiltration with effector CD8^+^/CD44^+^/CD62L^–^ T cells (**A**). Anti-PD-1+poly(I:C) significantly increases percentage of memory CD8^+^/CD44^+^/CD62L^+^ T cells in lymph nodes (**B**). Anti-PD-1+poly(I:C) increases the percentage of CD4^+^FoxP3^+^ Tregs in the spleen (**C**). Data are represented as mean ± SEM. All experiments repeated in triplicate with ≥5 mice per arm. *P*-values were determined by ANOVA, and, ^*^denotes statistical significance (*p* < 0.05).

Next we investigated what effect DC activation had on various lymphocyte populations. In tumor infiltrating lymphocytes (TILs), we found a statistically significant decrease in CD4^+^/FoxP3^+^ Tregs in mice treated with anti-PD1+poly(I:C) had a statistically significant decrease in percentage of Tregs when compared to control (Figure 2A. *p =* 0.0023). We also observed a trend towards increase in CD8^+^/CD44^+^/CD62L^−^ Teff, which was more marked in the combination therapy group (Figure 2A, *p =* 0.20). In the lymph node there was a significant increase in central memory T cells (CD8^+^/CD62L^+^/CD44^+^) in the group that received the combination treatment as compared to control mice (Figure 2B, *p =* 0.021). There were no differences in Teff and Tnaive (CD8^+^/CD62L^+^). In the spleen, the only statistically significant change observed was an increase in the Treg population in mice treated with anti-PD1+poly(I:C) as compared to control and anti-PD1 with a trend in increase in the mice treated with poly(I:C) alone (Figure 2C, *p =* 0.0003).

**Figure 3:**
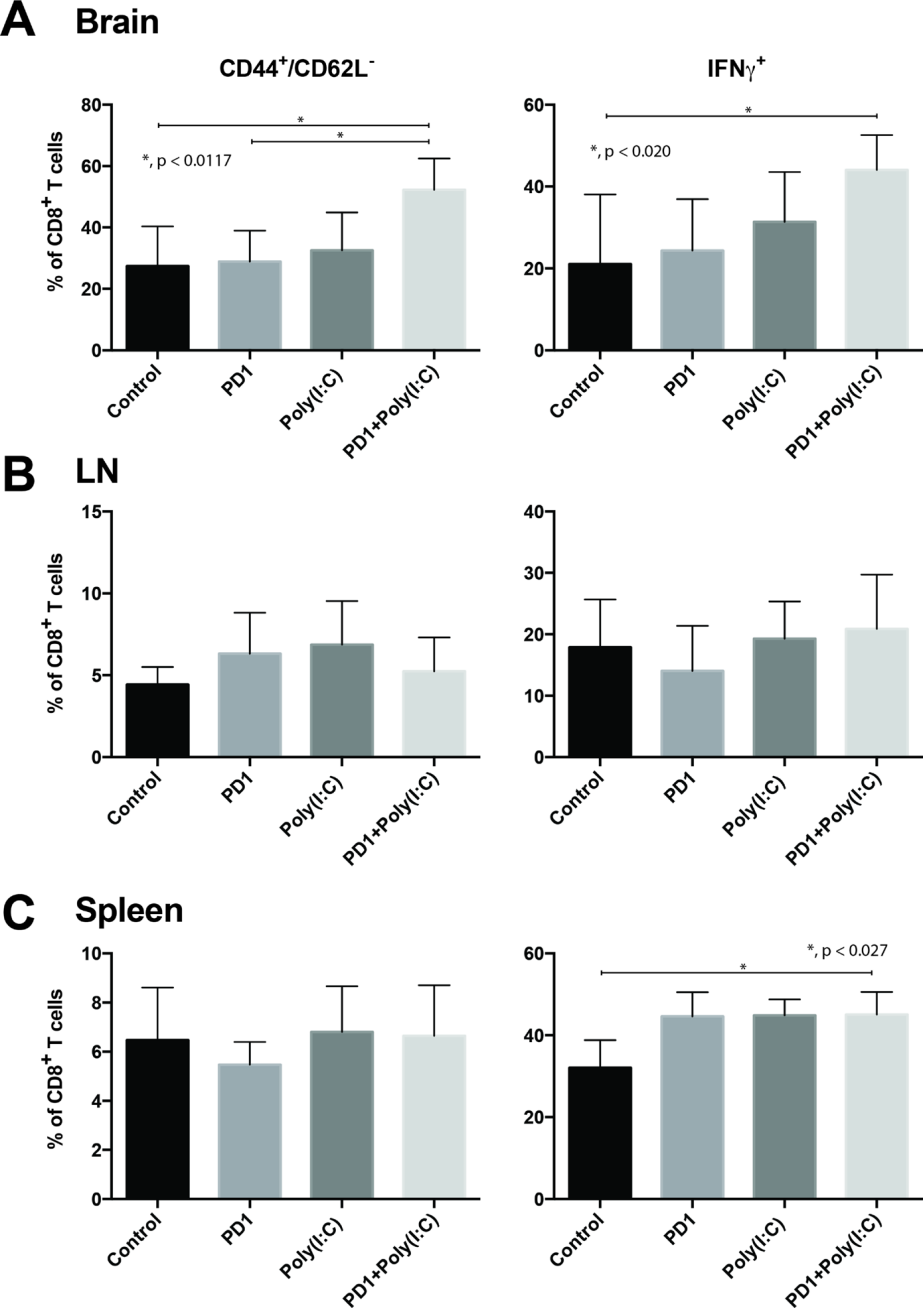
TLR3 agonist combined with PD-1 blockade promotes tumor infiltration of effector CD8+/CD44+/CD62L-/IFN-γ T cells Bar charts of T cells isolated from tumor bearing brains (**A**), deep cervical lymph nodes (**B**), and spleen (**C**). Charts present expression of CD44, CD62L, and IFN-γ in CD8^+^ T cells. Data are represented as mean ± SEM. All experiments repeated in triplicate with ≥5 mice per arm. *P*-values were determined by ANOVA, and, ^*^denotes statistical significance (*p* < 0.05).

We evaluated IFN-γ production after stimulation with phorbol 12-myristate 13-acetate(PMA)/ionomycin in cells isolated from the brain tumors, draining lymph nodes, and spleen. We found that there was a significantly higher percentage of infiltrating Teff CD8 TILs in the tumors of mice treated with anti-PD1+ poly(I:C) as compared to control and anti-PD1 treated mice (Figure 3A, *p =* 0.0117). Following *in vitro* PMA/ionomycin stimulation of harvested TILs there was a significantly higher percentage of cells expressing IFN-γ in the combination treatment group (Figure 3A, *p =* 0.020). There were no statistically significant differences in cells expressing TNF-α and cells co-expressing TNF-α/IFN-γ (data not shown). In the lymph node there were upward trends on CD8+ T cells expressing TNF-α and IFN-γ but there were no statistically significant differences (Figure 3B). The only statistically significant difference in the CD8+ T cells in the spleen was the increase in percentage of IFN-γ producing cells in the combination group (Figure 3C, *p =* 0.027). This suggests that treatment with anti-PD-1 and poly(I:C) induces a cytotoxic effector function in tumor-infiltrating CD8 T cells.

### Activation of antigen presenting cells in addition to anti-PD-1 therapy boosts survival and establishes a memory response

**Figure 4:**
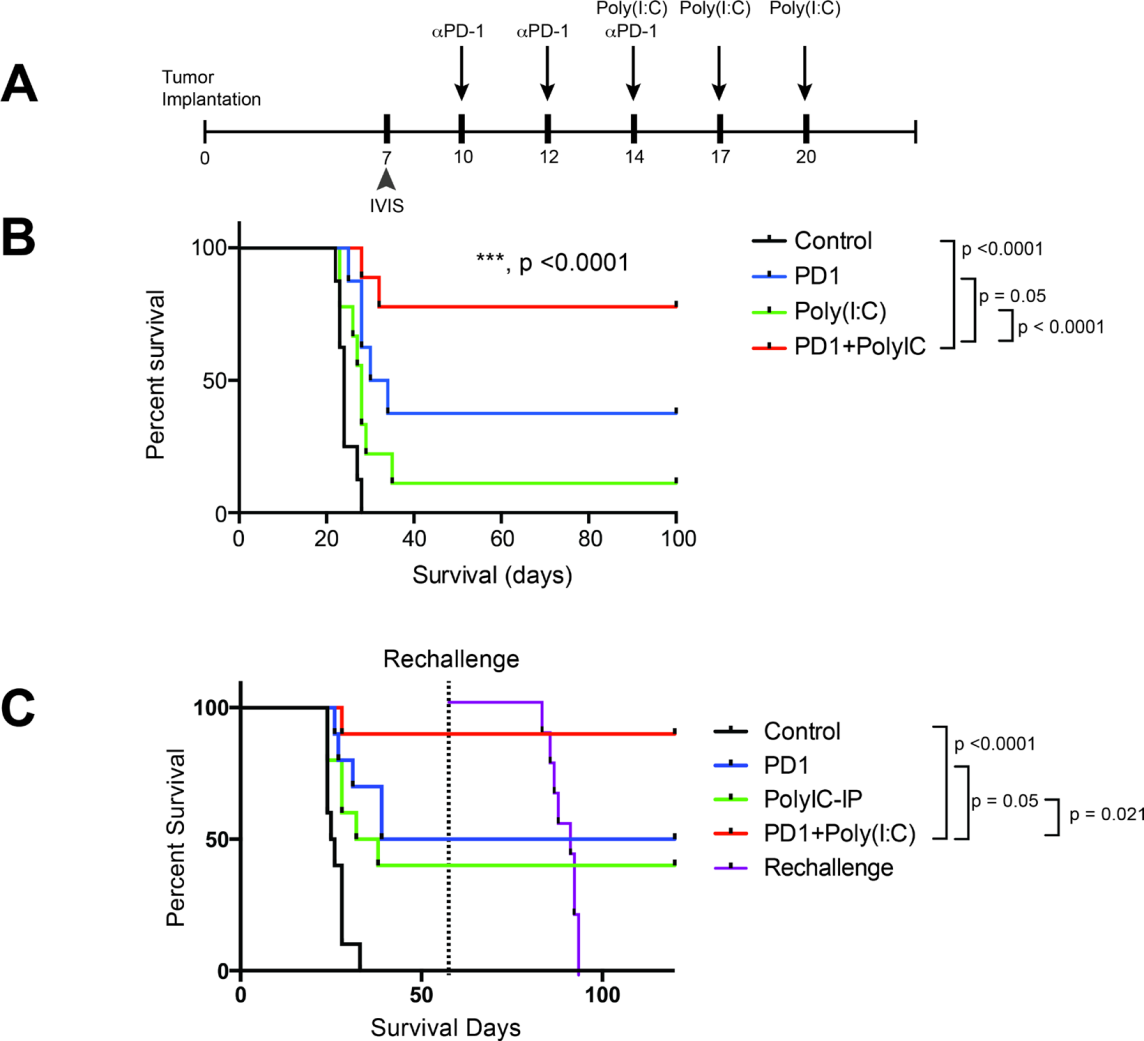
TLR3 activation + anti–PD-1 demonstrates superior survival profile compared to monotherapy regimens (**A**) Treatment schedule for survival experiment. (**B**) anti-PD-1+poly(I:C) treatment provides a significant increase in survival as compared to anti-PD-1 alone (*p* = 0.05). (**C**) In a separate experiment the same survival benefit was seen. Long-term survivor mice were rechallenged at day 60 by injecting 260,000 GL261-Luc cells in the cerebral hemisphere contralateral to the initial injection and their survival was tracked along with newly injected control mice. All newly injected control mice developed tumor and died whereas the long-term survivors did not. All experiments repeated in duplicate with ≥10 mice per arm. ^*^denotes statistical significance (*p* < 0.05) in survival.

As previously shown, therapy with a TLR3 agonist leads to DC activation and increased infiltration of Teff in the tumor, in addition to a decrease in tumor-infiltrating Tregs. We hypothesized that treatment with TLR3 agonist in addition to anti-PD-1 blockade would lead to improved survival in a preclinical orthotopic GBM mouse model. We assessed survival of untreated mice (Control), mouse treated with anti-PD-1 alone, Poly(I:C) alone, and anti-PD-1+Poly(I:C) (Figure 4A). Tumor presence was assessed at day 7 via bioluminescence imaging to ensure comparable tumor burden between groups. Poly(I:C) alone (median survival of 28 days) provided a slight survival benefit as compared to the control (median survival of 24 days) group (*p =* 0.032). Anti-PD-1 therapy (median survival of 32 days) resulted in a significant survival benefit when compared to control (*p =* 0.0007). The combination of anti-PD-1+poly(I:C) (did not reach median survival due to long-term survivorship) resulted in the greatest survival benefit when compared to control, anti-PD-1, or Poly(I:C) therapies (*p <* 0.0001) (Figure 4B). Additionally, we investigated whether dual therapy resulted in formation of a memory response. Long-term survivors were re-challenged in the contralateral hemisphere. While all the control (naïve) mice developed tumors, no long-term survivors developed tumors. None of the long-term survivors died, whereas the control mice showed the same short survival pattern as in the previous experiment (Figure 4C). Interestingly, only the combination of anti-PD-1+poly(I:C) treatment led to a significant increase in generation of memory T cells in CNS draining lymph nodes (Figure 2B). This data suggests that TLR3 agonist and PD-1 blockade leads to an effective activation of antigen presentation with formation of Teff response and generation of central memory T cells showing formation of effective anti-tumor immune memory.

### Survival benefit following treatment with TLR3 agonist is dependent on dendritic cells

It is well known that cross-presentation of antigen is performed mainly by CD8^+^ dendritic cells and that this process is important for the generation of a cytotoxic T cell responses against exogenous antigens as well as antigens found in tumorigenesis [12, 19]. We hypothesized that the increase in survival was due to activation of antigen presentation and to test this we depleted cross-presenting DCs [19]. The cytochrome C model for depletion of cross-presenting DCs was chosen because of its specificity. Genetic models such as CD11c-DTR mice are not specific to cross-presenting DCs, they have also shown that there is depletion of CD11b^+^ F4/80^+^ cells and depletion of these populations in this genetic model leads to expansion of a population of CD64^+^ monocytes [20]. Additionally, there is an expansion of a population of monocytes that express genes involved in TLR signaling, which may have interfered with our adminsitration of TLR3 agonist Poly(I:C) [20].

**Figure 5:**
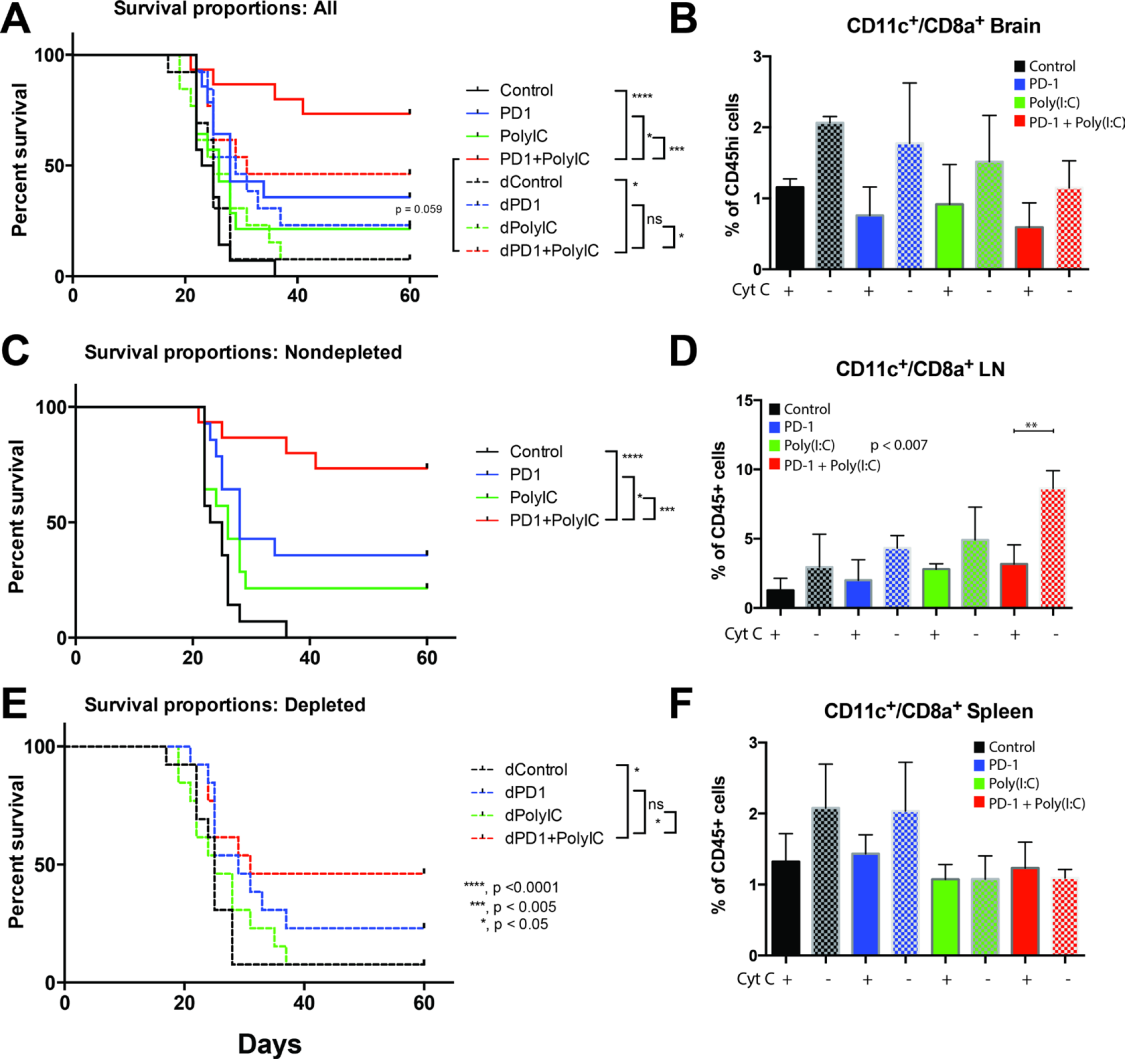
Depletion of cross-presenting DCs abolishes the survival benefit of TLR3 activation + anti–PD-1 Cross-presenting DCs were depleted by intravenous injection of equine cytochrome c. (**A**) Kaplan-Meier curve with control, anti-PD-1, Poly(I:C), and anti-PD-1+poly(I:C) treatment groups in non-depleted mice (solid lines) and depleted mice (dotted lines). The survival benefit of combination therapy is lost when DCs are depleted (**B**) Bar chart showing the percentage of CD11c^+^/CD8a^+^ cells isolated from the brain in tumor bearing mice in different treatment groups. Solid bars = depleted mice, checker bars = non-depleted mice). (**C**) Kaplan-Meier curve with control, anti-PD-1, Poly(I:C), and anti-PD-1+poly(I:C) treatment groups in non-depleted mice. (**D**) Bar chart showing the percentage of CD11c^+^/CD8a^+^ cells isolated from the lymph node in tumor bearing mice in different treatment groups. Solid bars = depleted mice, checker bars = non-depleted mice). Interestingly, the anti-PD-1+poly(I:C) treated mice showed a significant increase in CD11c^+^/CD8a^+^ cells in lymph nodes that was eliminated by cytochrome c treatment. (**E**) Kaplan-Meier curve with control, anti-PD-1, Poly(I:C), and anti-PD-1+poly(I:C) treatment groups in depleted mice. (**F**) Bar chart showing the percentage of CD11c^+^/CD8a^+^ cells isolated from the spleen in tumor bearing mice in different treatment groups. Solid bars = depleted mice, checker bars = non-depleted mice). All groups had 15 mice per arm. ^*^denotes statistical significance (*p <* 0.05) in survival. Data are represented as mean ± SEM.

Administration of cytochrome c resulted in partial depletion of DCs in deep cervical lymph nodes, this difference was statistically significant in the group of anti-PD-1+Poly(I:C) (*p =* 0.007) (Figure 5D). Similar reduction in DCs was seen in brain and spleen ( Figure 5B and 5F). Partial DC depletion resulted in a loss of the survival benefit achieved by combination of anti-PD-1 therapy with Poly(I:C) and there was no added benefit of combination therapy against anti-PD-1 alone (Figure 5A and 5E). Mice with tumor from each group were imaged with bioluminescence 3 times during the experiment: one at day 7 (Supplementary Figure 1A) to ensure equivalent tumor burden between groups, one at day 21 and another time at day 35 to determine tumor clearance. As shown in Supplementary Figure 1B, only 50% of the mice that received cytochrome c and were in the anti-PD1+Poly(I:C) therapy group were able to clear tumor whereas mice in the anti-PD1+Poly(I:C) treatment group that did not receive cytochrome c had the highest rate of tumor clearance at day 35 (79% of mice cleared tumor at day 35) (Supplementary Figure 1C). This data emphasizes the importance of cross-presenting DCs in the formation of an anti-tumor immune response. Additionally, it shows that the benefit of the combination of anti-PD-1 and poly(I:C) is related to the activation of antigen presentation.

### Tumor infiltration and proliferation of adoptively transferred tumor antigen-specific CD8 T cells is enhanced by DC activation using TLR3 agonist

#### Draining lymph nodes may be the site of antigen presentation.

**Figure 6:**
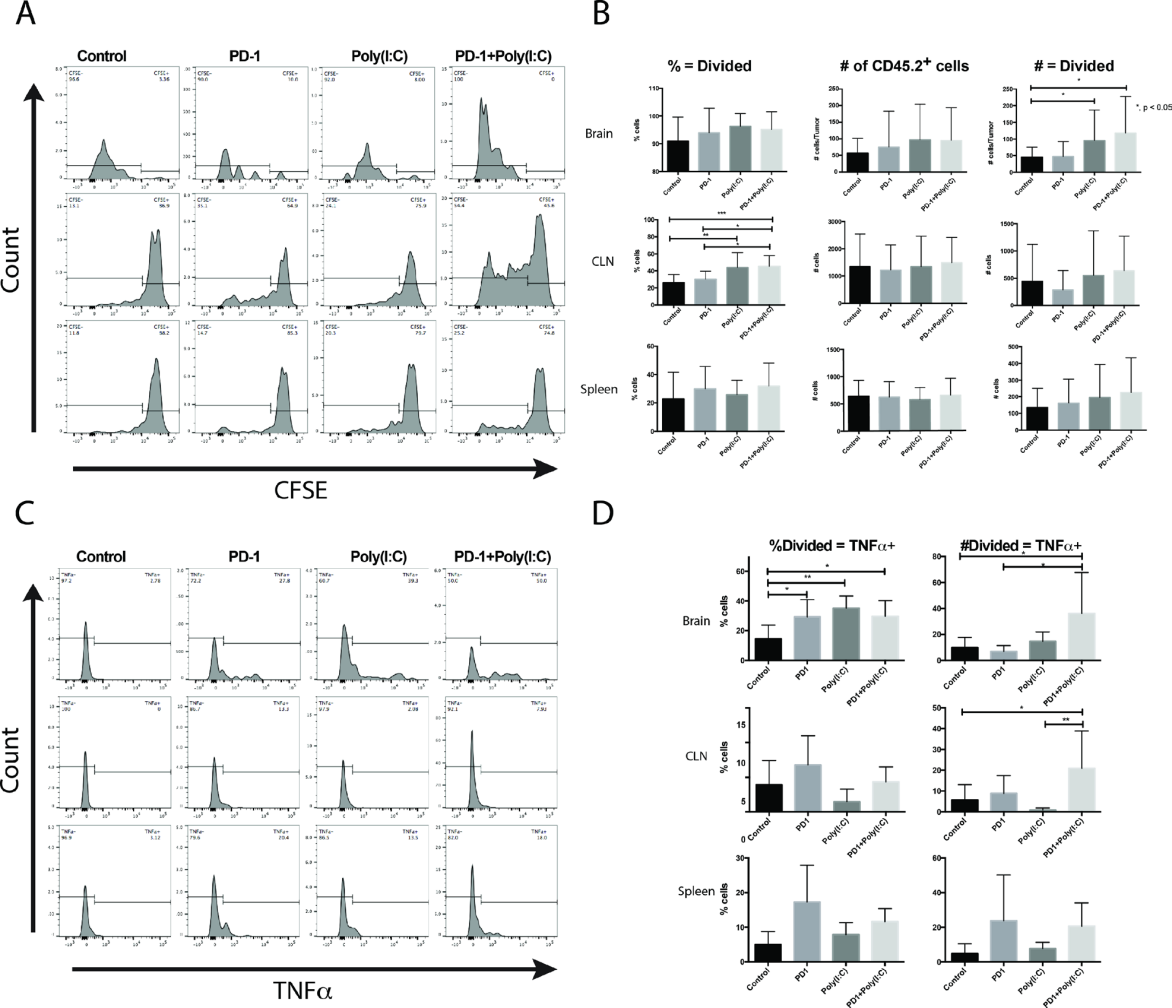
Tumor-specific CD8^+^ T cells undergo more divisions and produce more TNF-α when mice are treated with anti-PD-1 and Poly(I:C) (**A**) Representative histograms of CFSE in OT-1 T cells. (**B**) Summary bar charts showing percentages and numbers of OT-1 T cells undergoing more than 1 division in the brain, lymph node, and spleen. (**C**) Representative histograms of CFSE labeled OT-1 cells gated on cells undergoing more than 1 division and plotted against production of TNF-α. (**D**) Summary bar charts showing percentages and numbers of divided OT-1 T cells producing TNF-α in the brain, lymph node, and spleen. Data are represented as mean ± SEM. All experiments repeated in triplicate with ≥5 mice per arm. *P*-values were determined by ANOVA, and, ^*^denotes statistical significance (*p <* 0.05).

To determine the effects of antigen presentation enhancement on T cell function during an anti-tumor immune response and to gain information on the site of antigen presentation we implanted GL261-Ova cells expressing the Ovalbumin antigen (SINFEKL) in the brain of mice in each treatment group. On day 10 after tumor implantation, 3 × 10^6^ OT-1 CD8 T cells were labeled with CFSE (Carboxyfluorescein succinimidyl ester) and adoptively transferred into tumor bearing wild-type mice. OT-1 cells isolated from the brain tumors appeared to be terminally divided in all the groups (Figure 6A). OT-1 cells isolated from the lymph nodes displayed a range of cells with few to no divisions as well as cells that had undergone multiple divisions. In mice treated with anti-PD-1+Poly(I:C), OT-1 cells had undergone multiple divisions (Figure 6A). The OT-1 cells recovered from lymph nodes of Poly(I:C) and anti-PD-1+Poly(I:C) groups had more divisions than the cells recovered from lymph nodes from control or anti-PD-1 treated mice (Figure 6A and 6B). We also found that there was a significantly higher number of adoptively transferred cells infiltrating the brain tumor in the Poly(I:C) and anti-PD-1+Poly(I:C) treatment groups as compared to control mice and anti-PD-1 treated mice (Figure 6B, *p <* 0.05) and these cells showed evidence of division (Figure 6A).

To evaluate if there was a difference in effector function between groups we determined the number of divided cells able to produce TNF-α. TILs isolated from brain tumor-bearing mice treated with anti-PD-1, Poly(I:C), and anti-PD-1+Poly(I:C) had a significantly higher percentage of OT-1 divided cells that produced TNF-α. The actual number of divided OT-1 cells that produced TNF-α was significantly higher in mice treated with anti-PD-1 + poly(I:C) as compared to mice in the control, anti-PD-1, and poly(I:C) groups (Figure 6D). In lymph nodes, there was also a statistically significant increase in the divided OT-1 cells producing TNF-α, suggesting that antigen presentation may also be occurring in lymph nodes (Figure 6D). There were no significant changes in OT-1 cells recovered from the spleen. These results suggest that activation of antigen presentation with poly(I:C) results in increased formation of antigen specific effector T cells, that higher numbers of these cells infiltrate the brain tumors and that they divide in tumor draining lymph nodes, as a potential site for antigen presentation.

### Treatment with anti-PD-1 and TLR3 agonist increases expression of PD-1 ligands in myeloid cells

Because activating TLR3 receptors activates interferon secretion by dendritic cells [21–24] and interferon signaling results in changes in the expression of PD-1 ligands we sought to characterize the expression of PD-L1 and PD-L2 in the different sets of myeloid cells infiltrating the brain tumors and also in the peripheral lymphoid organs [5, 25].

We began by characterizing the expression of PD-L1 and PD-L2 in myeloid cells isolated from untreated tumor-bearing mice. It was surprising to find that there were striking differences in the mean fluorescence intensity (MFI) between the different types of myeloid cells analyzed. In brain tumor infiltrating myeloid cells, for PD-L1 microglia had the lowest MFI (Supplementary Figure 2A). We then evaluated the different DC populations into migratory, non-migratory, and CD8a^+^ DCs. and found that migratory DCs had by far the highest MFI for PD-L1(*p <* 0.0001). Migratory DCs had a significantly higher PD-L2 MFI than the rest of the myeloid cell populations studied (*p =* 0.0007). These findings were also encountered when studying the same cells in lymph nodes and spleen. (Supplementary Figure 2B and 2C).

When we compared expression of PD-L1 in myeloid cells infiltrating the brain tumor between the different treatment groups the only statistically significant difference was an increase in PD-L1 MFI in the macrophages of mice treated with anti-PD-1 (*p =* 0.029)(Supplementary Figure 3A). There were no significant changes in percentage of cells expressing PD-L1 between the different treatment groups, however, macrophages and non-migratory DCs had the highest percentage of PD-L1 expression (Supplementary Figure 4A).

In deep cervical lymph nodes, a large percentage of macrophages, migratory DCs, and non-migratory DCs expressed PD-L1 in control conditions (Supplementary Figure 4B). The percentage of PD-L1 expressing macrophages was significantly increased in poly(I:C) treated mice as compared to control and anti-PD1 treated mice (*p =* 0.0009). The percentage of PD-L1 expressing macrophages in anti-PD-1+poly(I:C) treated mice increased slightly but was not statistically significant. The percentage of migratory DCs expressing PD-L1 was significantly increased in mice treated with poly(I:C) and anti-PD-1+poly(I:C) as compared with control and anti-PD-1 group (*p <* 0.0001) (Supplementary Figure 4B). In non-migratory DCs the only treatment that significantly increased the percentage of PD-L1 expressing cells was the anti-PD-1+poly(I:C) therapy group (*p =* 0.0062).

When we evaluated the expression of PD-L1 using MFI values we found that in macrophages and migratory DCs, the MFI expression of PD-L1 was highest in the anti-PD-1+poly(I:C) group and there was a significant decrease in MFI in the poly(I:C), anti-PD-1, and control in that order in the lymph node (*p <* 0.0001) (Supplementary Figure 3B). The same behavior was seen in migratory DCs where the highest MFI was seen in the anti-PD-1+poly(I:C) therapy group in the spleen (*p <* 0.0001) (Supplementary Figure 3C).

In the spleen, also a large percentage of macrophages, migratory DCs, and non-migratory DCs expressed PD-L1 in control conditions (Supplementary Figure 4C). The percentage of PD-L1 expressing macrophages, migratory, and non-migratory DCs was significantly increased in anti-PD-1+poly(I:C) treated mice as compared to control and anti-PD-1 treated mice (*p <* 0.05).

PD-L2 expression did not change significantly among treatment groups and different myeloid cells did not show differences in expression in any of the compartments (data not shown).

Taken together, these data suggest that PD-L1 is the main ligand expressed and regulated in myeloid cells in the setting of combinatorial immunotherapy with TLR3 agonists and anti-PD-1.

### Activation of myeloid cells through other non-TLR3 agonists results in survival benefit in a mouse glioma model

**Figure 7:**
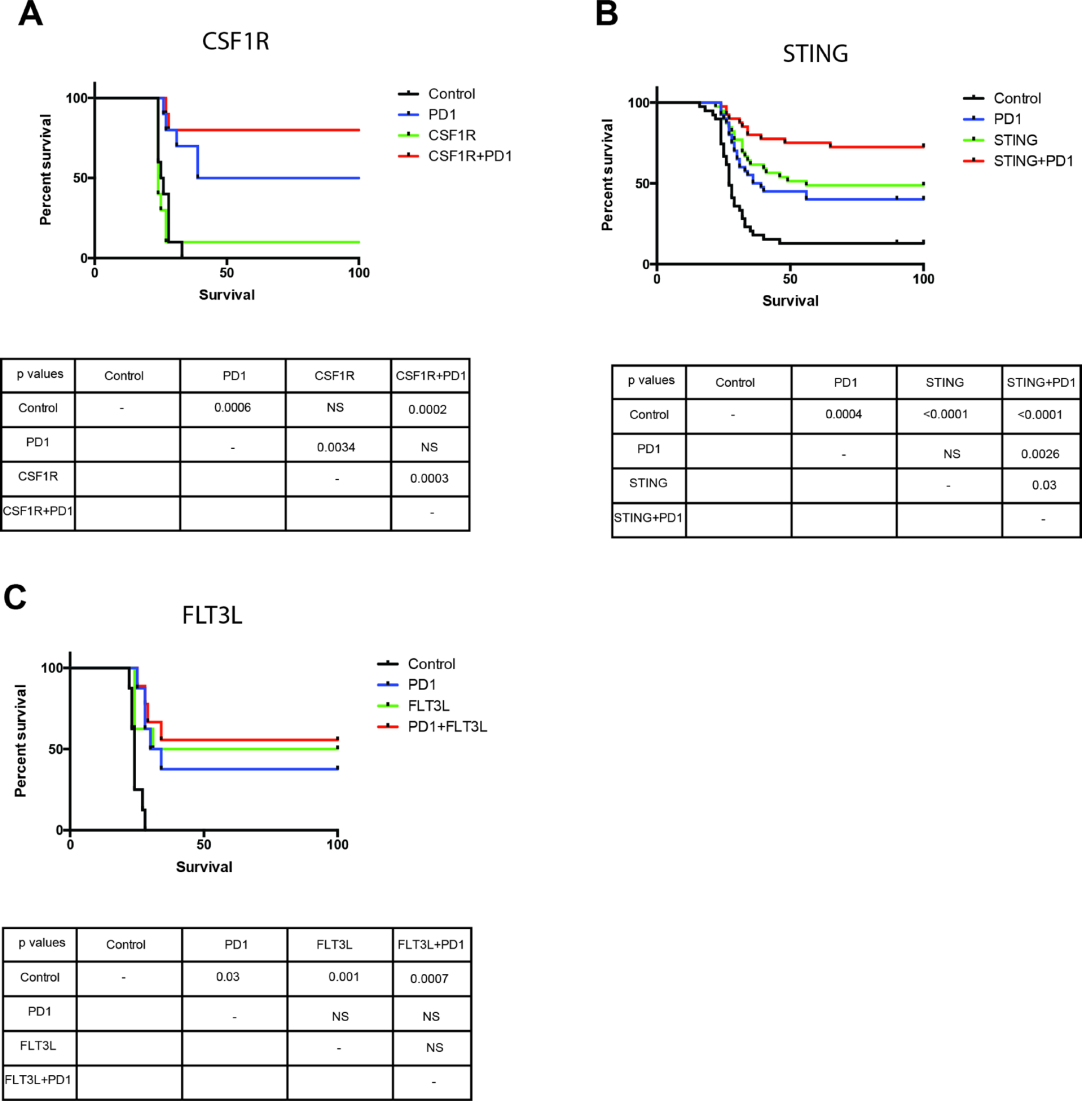
Myeloid cell activation through TLR independent agents results in survival benefit in a mouse glioma model Administration of CSF1R (**A**) and STING (**B**) with anti-PD-1 results in an enhanced survival benefit in a mouse model of glioma. Administration of FLT3L (**C**) with anti-PD-1 does not increase survival compared to monotherapy. All groups in CSF1F and FLT3L had 10 mice per arm, studies were repeated at least once. STING survival study had 40 mice per group.

We have tested other agents that activate the innate immune defense such as Sting, which is a TLR-independent pathway of innate defense triggered by nucleic acids. We tested the administration of FLT3L, a key factor in the differentiation of monocytes into dendritic cells [26], and showed that FLT3L administration provides a comparable survival benefit when compared to anti-PD-1, but there is no enhanced survival when FLT3L is administered with PD1. Additionally, anti-CSF-1R antibody was also tested to target tumor-associated macrophages and revert their inhibitory effect on the immune response [27]. CSF1R alone did not have a survival benefit but when combined with anti-PD-1 there was a significant enhancement of the survival benefit. We also tested the effect of STING, a TLR independent mediator of the innate immune response that is activated by cytosolic nucleic acids [28] and when combined with anti-PD-1 there was a significant enhancement in the survival benefit as compared with STING or anti-PD-1 alone (Figure 7). Together, the data from our survival experiments strongly suggests that targeting myeloid cells may be an effective strategy against GBM.

## DISCUSSION

GBM has been characterized as an immunosuppressive tumor [9, 29] that is able to alter the immune system by secreting immunosuppressive cytokines such as TGF-β, expressing immunosuppressive cell-surface factors such as CD95 and PD-1 ligands, and recruiting of immunosuppressive cells to the tumor microenvironment [29]. Further, response to immunotherapy will likely correlate with tumor infiltration by immune cells, which may depend on mutational burden, chromosomal and microsatellite instability, correlating with “hot” or “cold” tumors [30–32] We hypothesized that by targeting two separate but related immune mechanisms we could augment the anti-tumor immune response and extend survival in a mouse glioma model. To this end, we modulated antigen presentation though activation of TLR3 receptors targeting the myeloid compartment in conjunction with PD-1 blockade.

DC maturation is a critical event in the process of mounting an effective anti-tumor immune response. DC maturation and activation can be achieved through activation of TLR3 receptors among other stimuli. Poly(I:C) mimics viral double strand RNA and activates TLR3 receptors, promoting DC maturation and facilitating a T cell anti-tumor response [33]. TLR ligands mimic pathogen-induced maturation of DCs [34]. TLR3 receptors are expressed in a variety of immune cells such as DCs, NK cells, T cells, and macrophages, and microglia [21]. Activation of TLR3 leads to maturation of DCs and induction of interferon secretion resulting in amplification of the immune response [35]. Additionally, Poly(I:C) boosts anti-tumor T cell responses through generation of inflammatory cytokines and by decreasing T cell apoptosis [36–39]. TLR3 activation by using poly-ICLC has been used in clinical trials to treat patients with GBM alone and in conjunction with current standard of care with some benefit in survival when added to standard of therapy [40, 41]. In those studies, Poly-ICLC administration had a good safety profile with very few grade 3 adverse events and no grade 4 adverse events [40–43]. There are other studies supporting the safety of the use of Poly-ICLC in the treatment of glioma patients [44, 45]. Additionally, it has been shown that patients treated with Poly-ICLC combined with peptide-based vaccines and DC vaccines demonstrate an increase in survival [44, 45]. In several studies poly(I:C) has been used as adjuvant therapy to conventional anti-cancer therapy such as chemotherapy or as adjuvant to antigen-directed therapy such as DC and peptide vaccines [16, 21, 22, 24, 33, 38, 41, 43–47]. In our study the addition of poly(I:C) to anti-PD-1 therapy boosted an anti-tumor response and, in turn, survival in our glioma model (Figures 4 and 5). Our use of poly(I:C) in conjunction with CB, a therapy that does not rely on the identification of a specific antigen, is a fundamental change in the approach to use of poly(I:C) to treat cancer. The results obtained in the survival studies combined with the phenotypic changes in the immune cell populations expands the possible applications for poly(I:C).

DCs are activated by administration of poly(I:C) and can boost cross-presentation in *in vivo* models of cancer and as an adjuvant to anti-tumor vaccines [48, 49]. Poly(I:C) has been used multiple times as an adjuvant to anti-cancer vaccines resulting in reduction in tumor sizes as reviewed previously [21]. The vaccines tested in conjunction with poly(I:C) include DC based vaccines, tumor- associated antigen specific cell vaccines, and peptide vaccines [21] and multiple of the studies published show an increase in activated CD8^+^ T cells in the pre-clinical setting. In the clinical setting the use of poly(I:C) has been mainly done in trials evaluating its role as adjuvant of DC or peptide vaccines [21]. We focused on studying the effect of addition of poly(I:C) to CB to stimulate an effective anti-tumor response. Our results confirm previous findings of DC activation and increase in CD8^+^ T cell responses with the use of poly(I:C). Additionally we found that activating DCs in combination with CB led to improved survival in brain tumor bearing mice was dependent on the presence of cross-presenting DCs given that depletion of DCs resulted in abrogation of the survival benefit from combination therapy with anti-PD-1 and poly(I:C) (Figure 5). The importance of antigen cross-presentation has been recognized in order to generate an effective anti-tumor response in the context of vaccination [46]. Furthermore, activating antigen presentation bypasses the need to identify specific tumor antigens to generate an effective anti-tumor response.

The notion of the brain as an immune privileged environment poses a fundamental hurdle for immunotherapy to treat brain tumors [50, 51]. However, it has been recognized that this is not the case and that antigens in the brain are surveyed by the immune system [52–54]. Additionally, microglia, macrophages and dendritic cells present in the CNS act as potent antigen presenting cells [23, 55–58]. In our experiments we found that microglia and CD11c^+^ microglia did not change between treatment groups, however, their activation status was significantly lower in the anti-PD-1+poly(I:C) group, which is similar to the results observed in percentage of tumor infiltrating macrophages and migratory DCs. These data contrast to the results obtained when analyzing myeloid cells in the lymph node where there was an increased percentage of macrophages and dendritic cells, as well as their activation status in the combination therapy group (Figure 1B). This is in accordance with the model of DC maturation upon entry into secondary lymphoid organs where an immature DC collects antigen in the peripheral tissue, migrates to the secondary lymphoid organ, and becomes activated and expresses co-stimulatory molecules to activate T cells [34]. Other questions arise from these experiments and more studies are needed to determine whether antigen presentation activation in addition to immunotherapy leads to a higher number of antigens being presented or if there exists a more robust presentation of a limited pool of antigens.

The GBM tumor microenvironment is powerfully suppressive [29]. Patients with GBM have elevated levels of Tregs among many other immunosuppressive mechanisms [59]. In addition to DC activation, treatment with CB and Poly(I:C) had a beneficial effect by decreasing the percentage of tumor infiltrating Tregs (Figure 2A). It is well know that one of the mechanisms that tumors use to induce tolerance is through the recruitment, expansion, and activation of Tregs [60, 61]. It is extremely interesting that the treatment with PD-1 alone and in combination with Poly(I:C) decreased the infiltration in the brain by Tregs. The decrease in Tregs in the tumor can be seen in the group of mice treated with PD-1, although this effect is not statistically significant, however, when the combinatorial therapy is administered the difference is significant compared to control. Anti-PD-1 therapy is known to activate effector CD8^+^ cells leading to secretion of IFN-γ which, hypothetically may decrease the number of infiltrating Tregs by suppressing tumor-induced Treg proliferation and recruitment, albeit this decrease was not statistically significant [60, 61]. Poly(I:C) promotes the secretion of IFN-γ by dendritic cells [21, 62], therefore when poly(I:C) was added to anti-PD-1, the increase in IFN-γ led to a statistically significant decrease in tumor infiltrating Tregs. Moreover, it has been shown that not all long-term survivors form effective immune memory [63]. Even though all the long-term survivors rechallenged with tumor in our study showed effective tumor immunity, the only group that showed a significant increase in memory T cells after treatment was the anti-PD-1+poly(I:C) group (Figure 2B). This effect has also been demonstrated in a preclinical melanoma model where TLR3 agonist treatment resulted in more effective immune memory formation [39].

Finally, we investigated the effects of this treatment strategy on the expression of PD-L1 and PD-L2 in myeloid cells and found an increase in the percentage of myeloid cells expressing PD-L1 in the brain tumor, lymph node, and spleen. There was no change in PD-L2 expression. It is noteworthy that brain tumor infiltrating migratory DCs were found to have the highest expression of PD-L1 and PD-L2 (Supplementary Figure 2), and that treatment with anti-PD-1+poly(I:C) increased the expression of PD-L1 in myeloid cells mainly in the lymph nodes and in the spleen, and to a lesser degree in the brain tumor (Supplementary Figure 4). This phenomenon can lead to impaired activation of T cells and impair cross-presentation [64]. Further examination is required, however, this did not affect the anti-tumor response and survival.

Immunotherapy is acquiring a central role in the treatment of cancer. Although standard of care for patients with GBM does not include immunotherapy, several clinical trials are underway. In order to achieve the best anti-tumor response for GBM patients with immunotherapy we may need to manipulate multiple parts of the immune system and enhance different processes simultaneously. Our results suggest that activation of antigen presentation provides an effective way to boost the antitumor immune response. Current trials have used the activation of antigen presentation as an adjuvant in vaccine trials, whereas combination with CB may be more effective for GBM patients. These results suggest that by enhancing the activation of the innate and adaptive immune systems a more potent anti-tumor immune response can be achieved.

## MATERIALS AND METHODS

### Mice and cell lines

Animal protocols were approved by the Johns Hopkins Animal Care and Use Committee.

Female 6–8 week-old C57BL/6J wild-type mice or LY5.2 mice were housed and maintained at the Johns Hopkins University Animal Facility. OT-1/CD45.2/Rag−/− mice were used as donors for adoptive transfer experiments. GL261-Luciferase (purchased from *Caliper Life* Sciences; Hopkinton, MA) and GL261-OVA (kindly donated by Dr. Ollin; University of Minnesota) cell lines were grown in Dulbecco’s Modified Eagle Medium (DMEM, Life) + 10% fetal bovine serum (FBS, Sigma-Aldrich) + 1% penicillin-streptomycin (Life) with the addition of 100 μg/mL G418 (Corning) selection media at 37° C. They were tested for Mycoplasma on December of 2016 and found to be negative using MycoAlert Mycoplasma Detection Kit from Lonza. Once received from Survival experiments were at least triplicates with 6 to 10 mice in each arm.

### Tumor model

Six o 8 week old female C57BL/6J mice (The Jackson Laboratories, Bar Harbor, ME), had gliomas established by injecting 130,000 GL261-Luciferase cells stereotactically into the left striatum (2 mm posterior to the coronal suture, 2 mm lateral to the sagittal suture, and 3mm deep to the cortical surface) on day 0 of the experiments, as previously described [65]. Mice were anesthetized with ketamine/xylazine (100 mg/kg ketamine, 10 mg/kg xylazine) and placed in the stereotactic frame. A midline incision was made exposing the skull. A burr hole was drilled over the striatum. 130,000 GL-261 Luciferase cells were implanted in the coordinates cited above. Mice were randomly assigned to treatment arms and presence of tumor was monitored by bioluminescent IVIS^®^ imaging (*In Vivo* Imaging System, Caliper Life Sciences, Hopkinton, MA) on post-tumor implantation day 7.

For survival experiments each treatment group had 5 to 15 mice. Survival experiments without DC depletion were repeated 4 times. The treatment groups were as follows: (1) Control (200 µL PBS PBS administered intra-peritoneally (IP) on treatment days as shown in Figure 1), (2) anti-PD-1 (200 µg IP on day 10, 12, 14), (3) Poly(I:C) (100 µg IP on days 14, 17, 20) and anti-PD-1+Poly(I:C) at the same doses and dates as above. All experiments were done in triplicates unless otherwise stated. Animals were euthanized according to humane endpoints including central nervous system disturbances, hunched posture, lethargy, weight loss, and inability to ambulate.

For rechallenge experiments, long term survivors were considered cured at day 60 if IVIS imaging was clear of tumor. Long-term survivors from each experimental group were re-challenged 60 days post implantation with 260,000 GL-261-luciferase cells injected intracranially in the contralateral hemisphere. Naïve mice were implanted in parallel as controls, and mice were followed with weekly IVIS imaging.

### Adoptive transfer experiments

CD8 cells for adoptive transfers were obtained as follows: RAG−/− OT-I CD45.2 mice (kindly donated by Dr. Drake’s lab, Johns Hopkins University) were anesthetized to harvest spleens and lymph nodes. Red blood cell lysis buffer was applied subsequently. Viable cells were counted. CD8 T cells were labeled with CFSE (Invitrogen). Cells were resuspended in PBS at 15x10^6^ cells/mL and then transferred by retro-orbital injection (3 × 10^6^ cells) 10 days after implantation of GL261-OVA cells in the brain of 6 week old female B6.SJL-Ptprca Pepcb/BoyJ mice expressing the congenic marker CD45.1. Five to 6 days after adoptive transfer, brains, draining lymph nodes (DLN), and spleens were collected and homogenized. TILs were isolated using Percoll (Sigma) density gradient centrifugation per manufacturer instructions.

Cells were isolated and stimulated with 2 mmol/L H-2Kb–restricted class I epitope SIINFEKL (OVA257–264) in the presence of GolgiStop (BDBiosciences) and then analyzed by flow cytometry.

### Immune cell Isolation for flow cytometry

Mice were lethally anesthetized for lymphoid organ harvest. Brain, lymph nodes (deep cervical), and spleens were harvested after transcardial perfusion with phosphate buffered saline (PBS). Solid organs were mechanically homogenized in Roswell Park Memorial Institute (RPMI) medium + 10% FBS + 1% penicillin-streptomycin, and filtered through a 100-**μ**m mesh cell strainer (BD Falcon). Red blood cells were lysed from spleen samples and washed with PBS. To isolate tumor-infiltrating lymphocytes (TILs), brains were harvested on post-implantation day 20–22. Brains were processed as described previously [66]. Briefly, brains were were treated with DNase I (Sigma) and collagenase type IV (BRAND) in RPMI with 1% FBS and dissociated using gentleMACS dissociator, filtered and resuspended in 5 mL 70% Percoll, layered below 30% Percoll and centrifuged at 2000 rpm for 20 mins at RT. Cell layer at the 30%/70% interface was collected and washed with PBS.

### Flow cytometry of tumor infiltrating immune cells and peripheral lymphoid organs

Lymphocytes were stained for the markers in Supplementary Table 1, fixed in 1:3 fixation/permeabilization concentrate: diluent mixture (Ebioscience) for 30 mins, and stained for FoxP3 in permeabilization buffer. For analysis of the myeloid compartment, cells were treated with Fc block (anti-CD16/32), washed and stained with Live-Dead Aqua and stained for CD45, CD11b, CD11c, F4/80, MHC class II, CD86, and Ly6G. Appropriate isotype controls were used. Nonviable cells were excluded by forward vs. side scatter analysis and Live-Dead Aqua (Invitrogen) staining.

For adoptive transfer experiments, cells were stimulated for 4 hours with OVA peptide and stained for CD45.2-PB (Biolegend), CD8a-BV605, IFNg-PE-Cy7, TNFa-APC, CD62L-PerCP-Cy5.5 and CD44-AF700 and analyzed by flow cytometry. Flow cytometry was carried out using a LSR II (BD Biosciences). Data were analyzed using FlowJo software (Tree Star, Ashland, OR). The majority of flow cytometry experiments were repeated a minimum of 3 times with 5–7 mice per group each time.

### Therapeutic antibodies and reagents

Anti-PD-1 (Hamster monoclonal antibodies against murine PD-1) was purified from hybridoma (G4) as previously described [67, 68]. The treatment dose was 200 μg/dose. Poly(I:C) LMW was purchased from InvivoGen (San Diego, CA) and administered at 100 μg/dose. FLT3L was administered at a dose of 10ug/dose daily for 10 days on day 10 after tumor implantation; it was kindly provided by Celldex Therapeutics (Hampton, NJ). CSF1R antibody was administered at a dose of 300 ug/dose at day 10, 13, 17, and 20 after tumor implantation; it was provided by Bristol-Meyers-Squibb (New York, NY). STING (CDN) was administered at 0.5 ug once intratumorally day 10 post-implantation. The treatment schedule was performed as shown in Figure 1.

### Depletion studies

As previously described, cytochrome c from equine heart (Sigma, Carlsbad, CA) was used for ablation of cross-presenting dendritic cells (DCs) [19]. By administering exogenous cytochrome c DC numbers were decreased (Figure 5). A survival study was performed using the same treatment groups and their duplicates with cytochrome c administration.

### Statistical analysis

Data were analyzed by two-tailed Student *t* test or ANOVA using GraphPad (La Jolla, CA) Prism software. Survival was analyzed by Kaplan-Meier method and compared by log-rank test. Comparisons between groups were presented as mean ± SEM. Values of *p <* 0.05 were considered statistically significant.

## SUPPLEMENTARY MATERIALS FIGURES AND TABLE


