# Supplementary material for: Dendritic cell activation enhances anti-PD-1 mediated immunotherapy against glioblastoma
Source: Oncotarget. 2018 Apr 17;9(29):20681–97. doi: 10.18632/oncotarget.25061 (PMC5945499; doi:10.18632/oncotarget.25061)
Supplement: Supplementary file 1 [file oncotarget-09-20681-s001.pdf]

Dendritic cell activation enhances anti-PD-1 mediated immunotherapy against glioblastoma

SUPPLEMENTARY MATERIALS

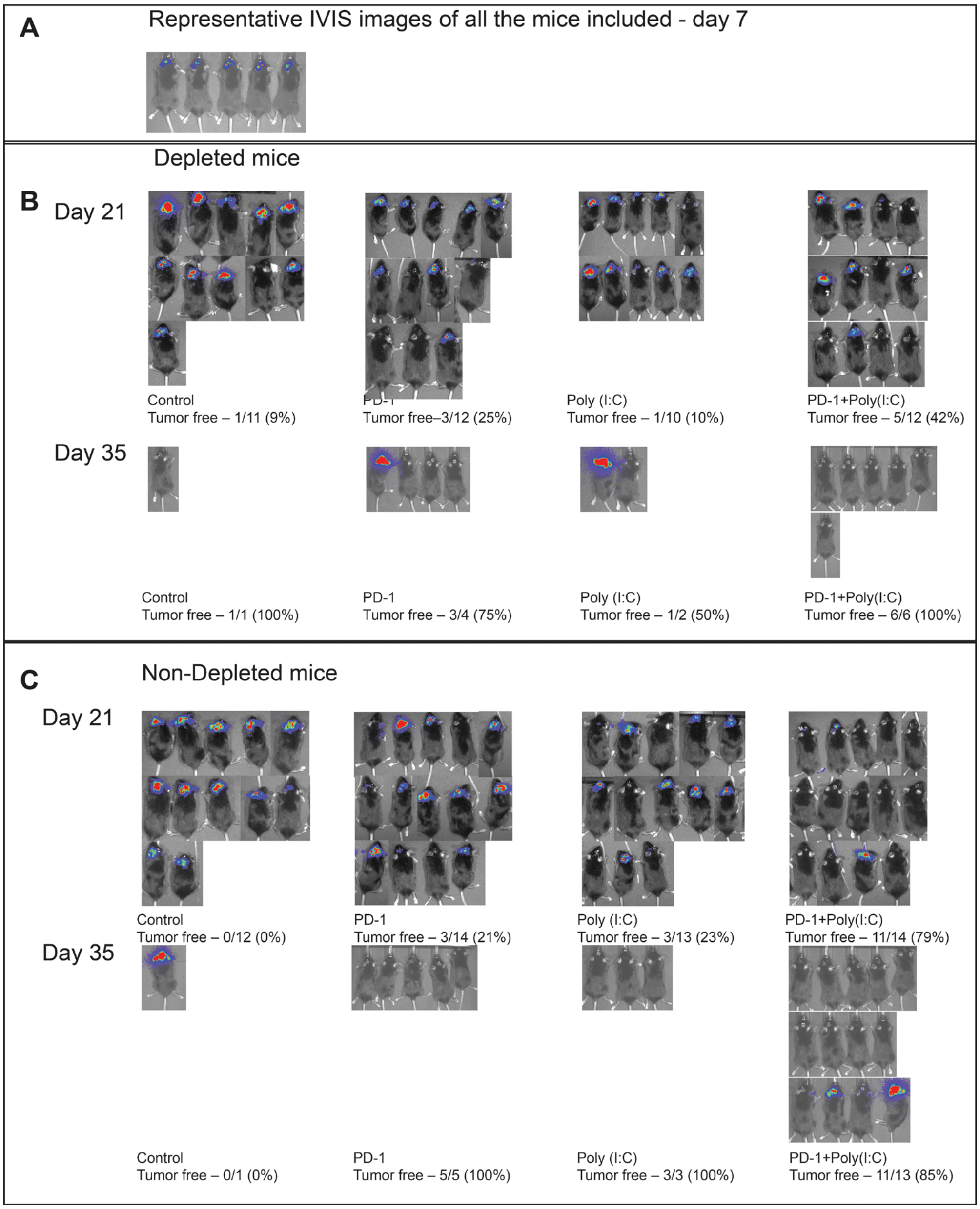

Supplementary Figure 1: Cross-presenting DC depletion decreases tumor clearance by mice treated with anti-PD-1+Poly(I:C). (**A**) Representative bioluminescence images of mice implanted with tumor at day 7. (**B**) Bioluminescence images of mice in different experimental groups treated with cytochrome c. (**C**) Bioluminescence images of mice in different experimental groups not treated with cytochrome c.

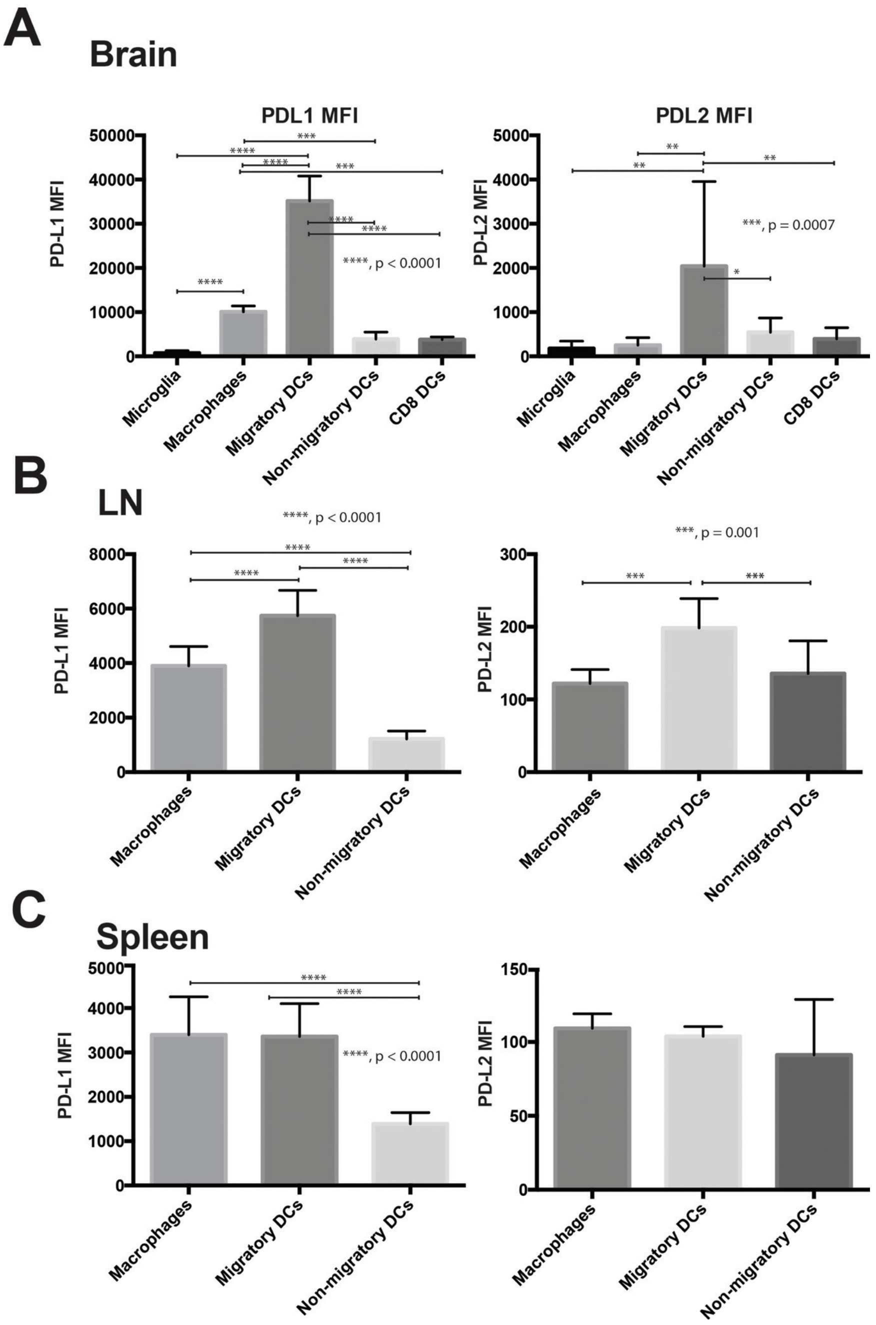

Supplementary Figure 2: Migratory DCs have the highest expression of PD-L1 and PD-L2 when compared to other myeloid cell populations in the brain tumor and in the lymph node. (**A**) MFI values of expression of PD-L1 and PD-L2 in myeloid cells isolated from brain tumors. (**B**) MFI values of expression of PD-L1 and PD-L2 in myeloid cells isolated from lymph nodes of brain tumor bearing mice. (**C**) MFI values of expression of PD-L1 and PD-L2 in myeloid cells isolated from spleen of brain tumor bearing mice. Data are represented as mean ± SEM. All experiments repeated in triplicate with ≥5 mice per arm. *P*-values were determined by ANOVA, and, *denotes statistical significance ($p < 0.05$).

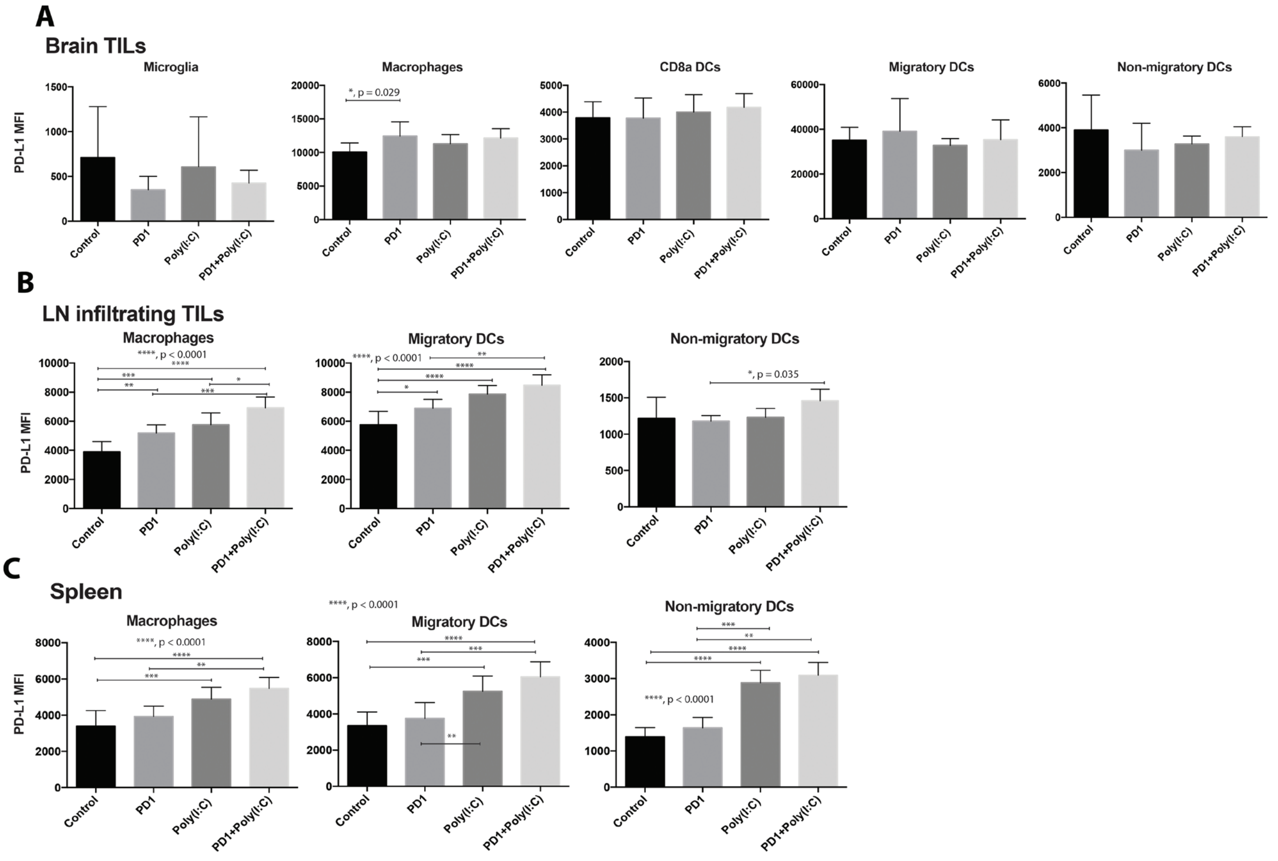

Suppplementary Figure 3: Treatment with anti-PD1+poly(I:C) increases expression of PD-L1 in myeloid cells in the lymph node and in the spleen. (**A**) Bar charts of MFI values of expression of PD-L1 in myeloid cells isolated from brain tumors in different treatment groups. (**B**) Bar charts of MFI values of expression of PD-L1 in myeloid cells isolated from lymph nodes of brain tumor bearing mice in different treatment groups. (**C**) Bar charts of MFI values of expression of PD-L1 in myeloid cells isolated from spleen of brain tumor bearing mice in different treatment groups. Data are represented as mean ± SEM. All experiments repeated in triplicate with ≥5 mice per arm. *P*-values were determined by ANOVA, and, *denotes statistical significance ($p < 0.05$).

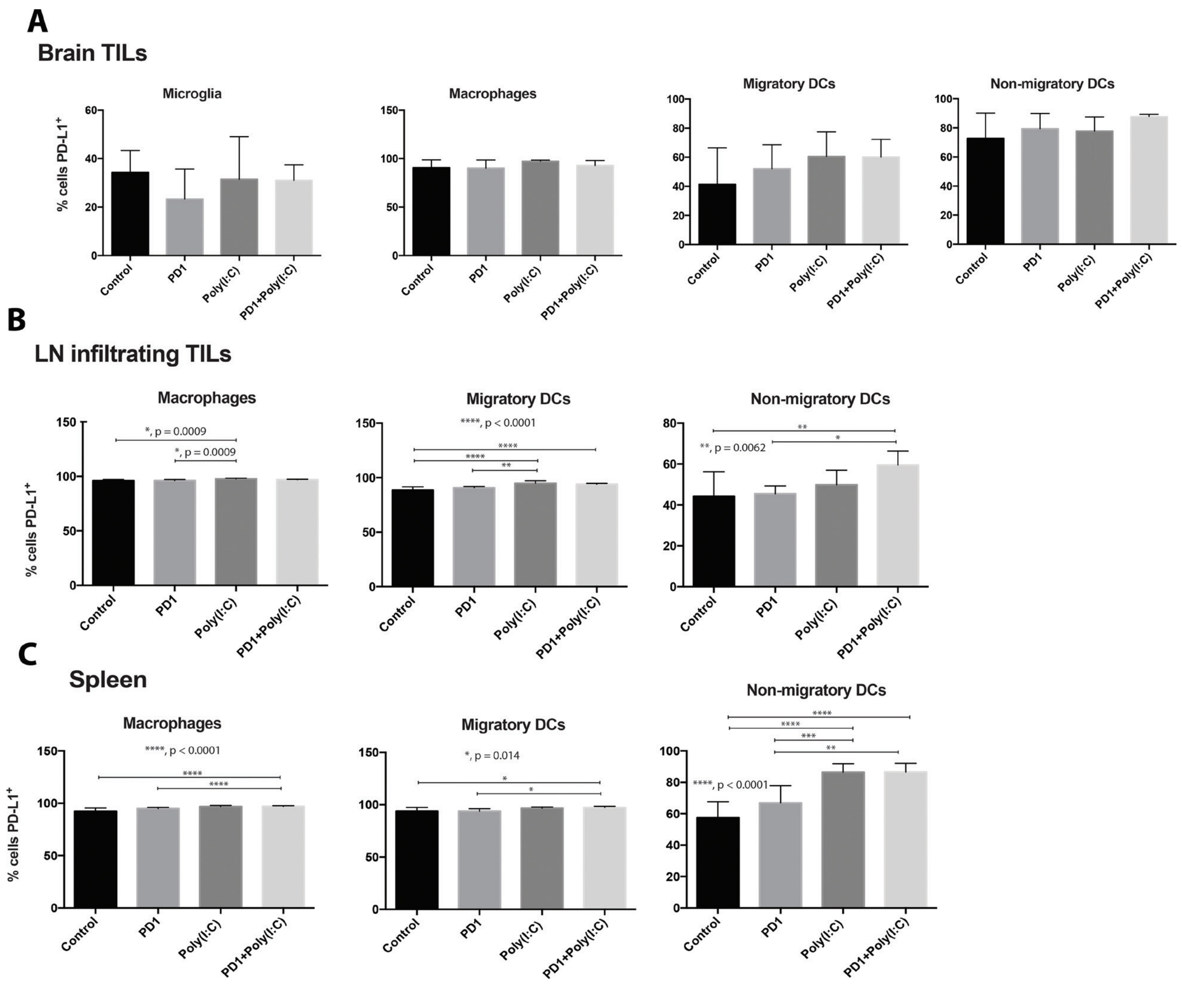

Supplementary Figure 4: DC activation and anti-PD-1 therapy increases expression of PD-L1 in myeloid cells. (**A**) Bar charts summarizing the effect of anti-PD-1, poly(I:C), and combination therapy on expression of PD-L1 of these in brain tumor infiltrating myeloid cells. (**B**) Bar charts summarizing the effect of anti-PD-1, poly(I:C), and combination therapy on expression of PD-L1 in brain tumor infiltrating myeloid cells in the lymph node. (**C**) Bar charts summarizing the effect of anti-PD-1, poly(I:C), and combination therapy on expression of PD-L1 in spleen myeloid cells. Data are represented as mean ± SEM. All experiments repeated in triplicate with ≥5 mice per arm. *P*-values were determined by ANOVA, and, *denotes statistical significance ($p < 0.05$).

Suppplementary Table 1: Antibody list

Antibody (flow)	Clone	Fluorophore	Dilution	Isotype	Clone
F 4/80	BM8	PE/Cy7	1:50		
CD3	145-2C11	FITC	1:500		
CD3	145-2C11	Pacific Blue	1:500		
CD4	RM4-5	FITC	1:500		
CD4	RM4-5	Pacific Blue	1:250		
F 4/80	BM8	PE/Cy7	1:50		
CD8	53-6.7	BV605	1:250		
FoxP3	FJK-16s	PE	1:200	Rat IgG2ακ	eBR2a
CD45	104	AF700	1:400	Rat IgG2βκ	RTK4530
CD45	104	Pacific blue	1:400	Rat IgG2βκ	RTK4530
CD11b	M1/70	AF700	1:500	Rat IgG2βκ	eB149/10H5
CD11c	HL3	FITC	1:500	Armenian Hamster IgG_1, λ2	G235-2356
PD-1	J43	PE/Cy7	1:200	Rat IgG2βκ	R35-38
TIM-3	RMT3-23	APC	1:200	Rat IgG2ακ	RTK2758
Lag3	Ebioc9b7w	PE	1:100	Rat IgG1 κ	RTK2071
CD44	IM7	AF700	1:250	Rat IgG2βκ	R35-38
CD62L	MEL-14	PerCP/Cy5.5	1:500	Rat IgG2ακ	RTK2758
IFN γ	XMG1.2	Pe/Cy7	1:1000	Rat IgG1κ	eBRG1
TNFα	MP6-XP22	FITC	1:500	Rat IgG1κ	R3-34
PDL1	10F.9G2	BV421	1:200	Rat IgG2βκ	R35-38
PDL2	TY25	PE	1:1000	Rat IgG2ακ	RTK4530
CX3CR1	SA011F11	APC	1:200	Rat IgG2ακ	RTK2758
I-A/I-E	M5/114.15.2	PerCP/Cy5.5	1:400	Rat IgG2βκ	R35-38
**Antibody (blocking)**	**Clone**	**Dilution**			
CD16/32	93	1:200			

mAbs were purchased from Biolegend, eBioscience, eInvitrogen, Life Technologies, or BD bioscience.
